# Earlier age at menarche is associated with higher diabetes risk and cardiometabolic disease risk factors in Brazilian adults: Brazilian Longitudinal Study of Adult Health (ELSA-Brasil)

**DOI:** 10.1186/1475-2840-13-22

**Published:** 2014-01-16

**Authors:** Noel T Mueller, Bruce B Duncan, Sandhi M Barreto, Dora Chor, Marina Bessel, Estela ML Aquino, Mark A Pereira, Maria Inês Schmidt

**Affiliations:** 1 Postgraduate Studies Program in Epidemiology, School of Medicine, Federal University of Rio Grande do Sul, Rua Ramiro Barcelos n. 2600, sala 414, Porto Alegre, Rio Grande do Sul, Porto Alegre 90035-003, Brazil; 2 Department of Epidemiology, Columbia University, New York, NY, USA; 3 Instituto de Saúde Coletiva, Universidade Federal da Bahia, Salvador, Bahia, Brazil; 4 Faculty of Medicine, Federal University of Minas Gerais, Belo Horizonte, Brazil; 5 National School of Public Health, Oswaldo Cruz Foundation, Rio de Janeiro, Brazil; 6 Division of Epidemiology & Community Health, School of Public Health, University of Minnesota, Minneapolis, MN, USA

**Keywords:** Puberty, Menarche, Diabetes, Cardiometabolic risk, Metabolic syndrome, Obesity, Brazil, Nutrition transition, Primordial prevention, Epidemiology

## Abstract

**Objectives:**

Early menarche has been linked to higher risk of type 2 diabetes in Western and Asian societies, yet whether age at menarche is associated with diabetes in Latin America, where puberty and diabetes may have different life courses, is unknown. We tested the hypothesis that earlier menarche is associated with higher diabetes risk in Brazilian adults.

**Methods:**

We used data from 8,075 women aged 35-74 years in the Brazilian Longitudinal Study of Adult Health (ELSA-Brasil) who had complete information on age at menarche, diabetes status, and covariates. Diabetes was defined based on self-reported physician diagnosis, medication use, and laboratory variables (fasting glucose, 2-hour glucose, and glycated hemoglobin). Poisson regression was used to generate risk ratios (RR) and 95% confidence intervals (CI).

**Results:**

Menarche onset < 11 years [vs. 13-14 years (referent)] was associated with higher risk of diabetes (RR = 1.34; 95% CI: 1.14-1.57) after adjusting for sociodemographic factors, maternal education, maternal and paternal diabetes, and birth weight. This persisted after further control for BMI at age 20 years and relative leg length. Additionally, among those not taking diabetes medications, earlier menarche [<11 years vs. 13-14 years (referent)] was associated with higher % glycated hemoglobin (*p* < 0.001), alanine aminotransferase (*p* < 0.001), triglycerides (*p* < 0.001), C-reactive protein (*p* = 0.003), waist circumference (*p* < 0.001), and BMI measured at baseline exam (*p* < 0.001).

**Conclusion:**

These findings support the hypothesis that earlier menarche is associated with greater risk for adult diabetes and cardiometabolic disease in the Brazilian context.

## Background

Type 2 diabetes has emerged over the past two decades as a major priority in the health agenda of the underdeveloped world [1,2]. Developing countries, while still suffering nutritional burdens of scarcity and infection, are faced with the challenge of managing increasing number of persons with type 2 diabetes and related micro- and macro-vascular complications [1,3]. In most low- and middle-income countries type 2 diabetes is now a leading cause of disease burden due to its long duration and devastating consequences with respect to quality of life and economic burden [4].

In Brazil, routine surveillance and periodic surveys on chronic diseases and their risk factors collected by the Ministry of Health indicate a considerable burden of diabetes [5]. In addition to the aging Brazilian population, there has been a rapid rate of migration from rural to urban environments [6]. Social, behavioral, nutritional, and environmental changes accompanying this transition are believed to underlie increases in fatness, insulin resistance, and type 2 diabetes [7].

Once diabetes manifests, its remediation through behavior change poses a formidable challenge [8,9], and, even if achieved, it may be too late to reduce incidence of cardiovascular events [10]. This emphasizes the need for prevention before type 2 diabetes and its risk factors develop (i.e., primordial prevention). Identifying valid markers of pubertal timing could pave the lifecourse avenue to primordial prevention of type 2 diabetes and its risk factors at the preclinical stage. Pubertal timing is influenced by genetic and environmental factors, including childhood nutrition and obesity [11-16]. In females, menarche is the most distinguishable pubertal marker. There is evidence for a trend towards earlier menarche in Brazil over the last several decades [17] that is not explained by childhood obesity [18].

Studies from Western countries [19-23] have reported that earlier age at menarche is associated with higher risk of type 2 diabetes. Two Asian studies [24,25] have found a similar association, while one has not [26]. No studies have examined this association in an economically and nutritionally transitioning Latin American population where contextual influences on age at menarche and type 2 diabetes may differ in important ways.

The aim of this manuscript was to examine age at menarche in relation to type 2 diabetes in Brazilian adults. Specifically, we will test the hypotheses that earlier age at menarche is associated with higher risk of diabetes, after adjustment for potentially confounding antecedents of pubertal timing and type 2 diabetes.

## Methods

### Study participants

The Brazilian Longitudinal Study of Adult Health (Estudo Longitudinal de Saude do Adulto or ELSA-Brasil) is a prospective cohort study designed to identify risk factors for diabetes and cardiovascular disease. The details of the study, including design, eligibility criteria, sources and methods of recruitment, have been described in detail elsewhere [27]. Briefly, the cohort comprises 8,217 female civil servants (from the total sample of 15,105 ELSA-Brasil participants), aged 35 to 74 years at baseline (2008-2010), who were sampled from 6 universities or research institutions (and, in a few instances, also of related educational or health institutions) located in different regions of Brazil: the University of São Paulo (*n* = 2,727); the Federal Universities of Minas Gerais (*n* = 1,642), Rio Grande do Sul (*n* = 1,172), Bahia (*n* = 1,189), and Espirito Santo (*n* = 554); and the Oswaldo Cruz Foundation (*n* = 933). All data for the current analyses were collected at baseline during initial interviews (~1 hour) and the first clinic visit (~5 hours). The study was approved by the Research and Ethics Committees of the institutions involved: Hospital de Clinicas de Porto Alegre, Universidade Federal do Rio Grande do Sul, Universidade Federal do Espírito Santo, Universidade Federal de Minas Gerais, Universidade Federal da Bahia, Universidade de São Paulo, Fundação Osvaldo Cruz.

For the current analyses, we excluded women with missing information for: diabetes status (*n* = 2), race/color (*n* = 74), age at menarche (*n* = 37), height (*n* = 3), sitting height (*n* = 2), or those who reported menarche onset < 8 (*n* = 7) or > 18 years (*n* = 11). To minimize inclusion of type 1 diabetes cases in our diabetes definition we excluded women who were diagnosed ≤ 30 years and used insulin as their first medication (*n* = 6). We created a dummy variable for missing birth weight (~14%) to compare the results including versus excluding participants with missing birth weight. Our final analytic sample comprised 8,075 women.

### Data collection

#### Exposure assessment

Age at menarche was assessed by the open-ended question, “At what age did you have your first menses?” Adult retrospective reports of menarcheal age have a moderately high (r = 0.79) correlation with original adolescent reports [28]. Height and sitting height (vertex of the head to the seated buttocks) were measured according to standard equipment and techniques. Leg length was determined by taking total height minus sitting height. Relative leg length (i.e., leg length-to-sitting height ratio) was calculated by taking leg length divided by sitting height.

#### Outcome assessment

A 12-hour fasting blood sample was drawn by venipuncture soon after the patient arrived at the baseline clinic visit. A 2-hour 75-g oral glucose tolerance test (OGTT) was administered only to participants without known diabetes. Glucose was measured by the hexokinase method (ADVIA Chemistry; Siemens, Deerfield, Illinois). Percent glycated hemoglobin (A1C) was measured using a high-pressure liquid chromatography (Bio-Rad Laboratories, Hercules, California), ad insulin with an immunoenzymatic assay (ELISA) (Siemens).

Diabetes status was classified using blood glucose measurements and self-reported information. A participant was considered to have previously diagnosed diabetes when answering, “yes” to either “Have you been previously told by a physician that you had diabetes (sugar in the blood)?” or “Have you used medication for diabetes in the past 2 weeks?” The remaining ones were evaluated for undiagnosed diabetes based on their laboratory values and then classified as having diabetes if they reached the threshold for fasting plasma glucose (≥ 126 mg/dL), 2-hour post load plasma glucose (≥ 200 mg/dL), or % A1C (≥ 6.5%).

Additional study outcomes included: alanine aminotransferase (ALT), measured by a modified International Federation for Clinical Chemistry (enzymatic) assay (ADVIA Chemistry), high-sensitivity C-reactive protein (CRP), measured by immunochemistry (nephelometry) (Dade Behring; Siemens), high density lipoprotein cholesterol (HDL-c), measured by enzymatic colorimetric assay (ADVIA Chemistry), and triglycerides, measured enzymatic colorimetric assay (glycerol phosphate peroxidase)(ADVIA Chemistry).

#### Covariate assessment

A comprehensive set of questionnaires, tests, and measurements was carried out to control for co-varying parameters. Age at baseline visit (years), race/skin color, educational achievement of the participant and their mother, parental history of diabetes, birth weight, polycystic ovary syndrome (PCOS), menopause, parity, oral contraceptive use, hormone therapy, smoking status, alcohol use, physical activity, and diet were ascertained by questionnaire. Body mass index (BMI) was calculated as weight (kg) divided by height (m) squared. Self-recalled weight was used to determine participants’ BMI at 20 years of age.

### Statistical analysis

All analyses were performed using SAS 9.2 (SAS institute, Cary, NC). We summarized the characteristics of the cohort using unadjusted means and standard deviations for continuous variables and percentages for categorical variables according to menarcheal age categories (<11, 11-12, 13-14, 15-16, and 17-18 years). The median age at menarche category, 13-14 years of age, was used as the reference group for regression models.

We used multivariable Poisson regression with robust variance [29] to address the main hypothesis of our study. This allowed us to estimate risk ratios (RRs) and 95% confidence intervals (CIs) for diabetes according to categories of menarcheal age. Multivariable models were contrasted in a series beginning with model 1 adjusted for study center and age at interview (years). Model 2 included further adjustment for socio-demographic background variables: race/skin color, and maternal education (no formal education, less than eighth grade, completed eighth grade but not high school, completed high school but no college, any college). We then additionally adjusted for maternal and paternal diabetes (model 3) and self-reported birth weight (<2500 g, 2500-4000 g, >4000 g, missing) (model 4). As we were interested in the age at menarche-diabetes association independent of early-adult BMI, in model 5 we further adjusted for BMI (based on self-reported weight) at age 20 years (the earliest measure of this variable). Lastly (model 6), we added relative leg length to determine if age at menarche is independent of this marker of early-life growth and developmental adequacy [30].

We evaluated effect measure modification in the final model by including cross-product terms between our exposures and age at interview (median split; < 55 vs. ≥ 55 years), race/skin color (black vs. white), maternal education (< high school vs. ≥ high school), birth weight (< 2500 vs. ≥ 2500 g), and BMI at 20 years (25 kg/m^2^ vs. ≥ 25 kg/m^2^).

In additional analyses, we used multivariable linear regression to assess age at menarche categories in relation to continuous outcome measures for % A1C, glucose, insulin, alanine aminotransferase, triglycerides, HDL-c, high sensitivity CRP, waist circumference, and BMI measured at baseline exam. For these analyses we excluded women taking diabetes medications in the two weeks prior to baseline exam. All statistical tests were two-sided and significance was defined at *p* < 0.05.

## Results

The mean (standard deviation) age at menarche for the 8,075 women eligible for this analysis was 12.7 (1.7) years of age. As seen from the participant characteristics according to age at menarche categories presented in Table 1, earlier menarche age was associated with younger age at enrollment, higher maternal educational attainment, higher prevalence of paternal diabetes, shorter leg length (but not sitting height), and higher BMI at age 20. Women from early and late menarcheal age categories were more likely to self-identify as black (color), be born prematurely, have mothers with diabetes, or have PCOS.

**Table 1:** Mean and standard deviation (unless otherwise noted) of baseline characteristics by age at menarche in women aged 35-74 years from the Brazilian Longitudinal Study of Adult Health (ELSA-Brasil)

	**Total**	**<11 years**	**11–12 years**	**13–14 years**	**15–16 years**	**17–18 years**
*n* (range)*	6943-8075	574-657	2737-3124	2720-3181	749-912	166-204
Age at enrollment	52.0 ± 8.8	51.4 ± 8.4	51.2 ± 8.9	52.3 ± 8.9	53.5 ± 8.7	53.9 ± 8.3
Race/skin color (% black)	17.9	19.6	18.2	16.9	18.1	22.1
Maternal education (% ≥ high school)	22.7	26.0	24.8	22.1	16.9	12.1
Participant education (% ≥ secondary)	54.5	53.9	58.4	54.8	43.8	40.2
Maternal diabetes (%)	19.9	22.2	20.0	19.4	19.2	21.6
Paternal diabetes (%)	13.6	19.6	14.9	12.0	10.9	10.8
Premature birth (%)	5.6	7.6	5.5	5.1	5.9	6.5
Low birth weight (%)	8.7	9.9	8.7	7.5	12.1	10.8
PCOS (%)	11.1	14.9	11.1	10.7	9.3	11.8
Post-menopause (%)	59.2	60.4	56.6	59.6	64.3	64.7
Five or more births (%)	9.8	9.9	9.6	9.4	11.5	11.3
Leg length (cm)	74.2 ± 4.4	73.0 ± 4.7	74.0 ± 4.4	74.5 ± 4.2	74.7 ± 4.3	75.7 ± 4.2
Sitting height (cm)	84.8 ± 3.7	84.7 ± 4.2	84.8 ± 3.9	84.8 ± 3.5	84.6 ± 3.5	84.4 ± 3.4
BMI (kg/m^2^) at age 20 years	20.9 ± 3.1	21.9 ± 3.7	21.1 ± 3.1	20.6 ± 2.9	20.4 ± 3.2	20.0 ± 3.1

*Abbreviations*: *PCOS* polycystic ovary syndrome.

*n varied for each continuous metabolic parameter included in the model, so the range of *n* is included in parentheses.

Approximately 16.5% of the women included in our analyses were classified as having diabetes. The association between age at menarche and diabetes after multiple levels of covariate adjustment is shown in Table 2. Age at menarche <11 years [vs. 13-14 years (referent)] was associated with diabetes after adjusting for study center, age, socioeconomic background, maternal and paternal diabetes, and birth weight (model 4, RR = 1.34; 95% CI: 1.14, 1.57). This association was modestly attenuated after further adjustment for BMI at age 20 years (model 5, RR = 1.26; 95% CI: 1.07, 1.49), and relative leg length (model 6, RR = 1.23; 95% CI: 1.04, 1.44). Further inclusion of participant education level (less than high school, completed high school but not college, completed college but not graduate school, graduate school plus), smoking status (never, former, current), alcohol use (yes/no), leisure time physical activity (light, moderate, hard), PCOS (yes/no), postmenopausal status (yes/no), and parity (0, 1-2, 3-4, 5+) did not materially alter the results (RR = 1.19; 95% CI: 1.01, 1.41). As these factors are likely to be on the exposure-outcome causal pathway (i.e., mediators), they were not retained in the final model (model 4). Adjustment for BMI at baseline–a major mediator on the causal pathway–did attenuate the menarche age–diabetes association (data not shown).

**Table 2:** Risk ratios (and 95% confidence intervals) for type 2 diabetes by menarcheal age in women aged 35-74 years from ELSA-Brasil

	**<11 years**	**11–12 years**	**13–14 years**	**15–16 years**	**17-18 years**
Cases/*n*	144/657	483/3121	513/3181	158/912	37/204
Model 1	1.43 (1.22-1.69)	1.02 (0.91-1.14)	1.0 (Ref.)	1.00 (0.86-1.18)	1.04 (0.77-1.40)
Model 2	1.44 (1.22-1.69)	1.03 (0.92-1.15)	1.0 (Ref.)	0.96 (0.82-1.13)	0.99 (0.73-1.33)
Model 3	1.35 (1.15-1.58)	1.01 (0.91-1.13)	1.0 (Ref.)	0.97 (0.83-1.13)	0.98 (0.73-1.32)
Model 4	1.34 (1.14-1.57)	1.01 (0.90-1.13)	1.0 (Ref.)	0.95 (0.81-1.11)	0.98 (0.73-1.31)
Model 5	1.26 (1.07-1.49)	0.99 (0.88-1.10)	1.0 (Ref.)	0.97 (0.82-1.13)	1.05 (0.79-1.41)
Model 6	1.23 (1.04-1.44)	0.98 (0.87-1.09)	1.0 (Ref.)	0.97 (0.83-1.14)	1.07 (0.80-1.44)

Model 1: adjusted for age at enrollment, and study center.

Model 2: adjusted for variables in model 1 and race/color, and maternal education.

Model 3: adjusted for variables in model 2 and maternal diabetes, and paternal diabetes.

Model 4: adjusted for variables in model 3 and birth weight.

Model 5: adjusted for variables in model 4 and BMI at age 20 years.

Model 6: adjusted for variables in model 5 and relative leg length.

We did not observe evidence that associations differed by age at interview (median split; <55 vs. ≥55 year), race/skin color (black vs. white), maternal education (<high school vs. ≥high school), birth weight (< 2500 vs. ≥ 2500 g), or BMI at 20 years (25 kg/m^2^ vs. ≥25 kg/m^2^). Conclusions from our analyses including participants with a dummy variable for missing birth weight were not materially different from those that excluded participants with missing birth weight.

In additional analyses among women not taking diabetes medications (*n* = 7,192– 7,396, depending on outcome), earlier menarche [< 11 years vs. 13-14 years (referent)] was associated with higher % A1C (*p* < 0.001), modestly associated with higher insulin concentrations (*p* = 0.07) and 2-hour post load glucose (*p* = 0.09; only analyzed in 7,100 women without previously diagnosed diabetes), but was not associated with fasting glucose (*p* = 0.68) (Table 3). Earlier menarche [< 11 years vs. 13-14 years (referent)] was also associated with elevated alanine aminotransferase (*p* < 0.001), triglycerides (*p* < 0.001), high sensitivity CRP (*p* = 0.003), waist circumference (*p* < 0.001), and BMI (*p* < 0.001) measured at enrollment (Table 3). Later age at menarche [17-18 years vs. 13-14 years (referent)] was associated with higher CRP (*p* = 0.01) but not with other cardiometabolic disease risk factors analyzed (all *p* > 0.05).

**Table 3:** Adjusted means and standard errors for indices of glycemic risk, cardiometabolic risk, and body habitus according to age at menarche in women aged 35-74 years in ELSA-Brasil not taking diabetes medications

	**<11 years**	**11–12 years**	**13–14 years**	**15–16 years**	**17–18 years**	**F-test *p***
*n (range)	536-579	2764-2864	2818-2930	804-831	178-185	
*Glycemic risk*						
% A1C	5.38 ± 0.03†	5.33 ± 0.01	5.30 ± 0.01	5.31 ± 0.02	5.31 ± 0.05	0.048
2-h post load glucose	130.2 ± 1.7	127.9 ± 0.7	127.0 ± 0.7	128.8 ± 1.4	128.7 ± 2.9	0.43
Fasting glucose	104.1 ± 0.6	104.4 ± 0.3	103.8 ± 0.3	103.6 ± 0.5	104.9 ± 1.1	0.54
Fasting insulin	8.3 ± 0.7	8.1 ± 0.3†	7.0 ± 0.3	7.1 ± 0.5	7.2 ± 1.2	0.057
*Cardiometabolic risk*						
ALT	26.9 ± 0.7†	23.3 ± 0.3	23.1 ± 0.3	22.2 ± 0.6	24.0 ± 1.2	<.001
Triglycerides	123.6 ± 2.8†	117.7 ± 1.3	115.6 ± 1.2	112.9 ± 2.3	114.7 ± 4.9	<.001
HDL-cholesterol	61.9 ± 0.6	61.5 ± 0.3†	62.5 ± 0.3	61.8 ± 0.5	61.6 ± 1.1	0.16
C-reactive protein	3.6 ± 0.2†	3.0 ± 0.1	3.0 ± 0.1	3.0 ± 0.2	3.9 ± 0.3†	0.005
*Body habitus*						
BMI	27.8 ± 0.2†	27.1 ± 0.1†	26.4 ± 0.1	26.5 ± 0.2	26.7 ± 0.3	<.001
WC	88.9 ± 0.5†	87.3 ± 0.2†	86.2 ± 0.2	86.8 ± 0.4	87.2 ± 0.8	<.001

*Abbreviations*: *A1C* glycated hemoglobin; *ALT* alanine aminotransferase; *HDL* high-density lipoprotein; *BMI* body mass index; *WC* waist circumference.

Adjusted for study center, age at enrollment, race/color, maternal education, maternal diabetes, paternal diabetes, birth weight, and BMI at age 20 years.

*n varied for each continuous metabolic parameter included in the model, so the range of *n* is included in parentheses; only those without previously diagnosed diabetes underwent 2-hour post load glucose testing (*n* = 7,100).

†*p* < 0.05 when compared to menarcheal age category 13-14 years (referent).

## Discussion

In this large cohort of Brazilian adults who were born and came of pubertal age before the economic and epidemiologic transition in Brazil, menarche onset less than 11 years was associated with higher risk of diabetes. This association remained after adjustment for potential confounders, including race, maternal education, parental diabetes, birth weight, relative leg length–a marker of early-life nutritional status [30]–and BMI at age 20 years.

Our study provides estimates of average age at menarche in a sample of Brazilian women participating in a large, free-living occupational cohort, born between 1934-1975 and raised during a period when the average gross domestic product of Brazil increased by 81% [31]. The earlier age at menarche observed with younger participants reflects the secular trend toward earlier age at menarche in Brazil [18]. While this trend may have been influenced by increases in childhood fatness, our study, corroborating evidence from others [20,22], suggests that the association between age at menarche and adult diabetes is not accounted for by early-life BMI. Our study adds an important piece of evidence to the literature base on this topic, showing that early menarche was associated with diabetes after full multivariable adjustment-including BMI at age 20 years-in Brazilian adults born and raised in an era when childhood stunting was more common (26.7%) than overweight (8.6%) [32].

Our findings largely align with literature on this topic from Asian cohorts that had low rates of childhood overweight/obesity and lived through rapid epidemiologic and nutritional transition after coming of pubertal age. Conway et al. found that in 69,385 middle-aged adult Chinese women from the Shanghai Women’s Health Study, a 1-year increment in menarcheal age was associated with a 5% lower risk of diabetes; this association was attenuated upon adjustment for BMI measured when participants were 40-70 years [24]. In the Singapore Chinese Health Study, menarche at or before 12 years of age (compared to 13-14 years) was associated with an 18% increased risk of diabetes even after adjustment for BMI self-reported when participants were aged 45-74 years [25]. In a study of 3,304 post-menopausal women from Fujian, China, age at menarche did not appear to be associated with diabetes, but the earliest category of menarche age in this analysis was 9-14 years thus potentially masking an association between diabetes and early menarche as defined by our study (<11 years) [26].

Studies from countries with longer-standing economic prosperity have also reported an association between earlier menarche and increased risk of diabetes [19-23]. Similar to findings from more recently transitioned populations, evidence from Western societies suggests the menarcheal age-diabetes association is independent of confounding by BMI. Findings from our study indicate the association between early age at menarche and diabetes is not explained by BMI at 20 years of age (the earliest measure of BMI in our cohort). These results are consistent with those from a British birth cohort [22], the EPIC-InterAct study [33], and the ARIC study [20], which found that BMI measured pre-menarche, at age 20, and at age 25, respectively, did not explain the age at menarche-diabetes association. Furthermore, of the studies mentioned herein, ours was the first to find that age at menarche was associated with diabetes independent of relative leg length, indicating these markers represent different aspects of early-life growth and development.

The mechanisms underlying the association between age at menarche and type 2 diabetes are likely myriad and interrelated. One extensively studied pathway is the triggering of puberty by adipocytes and related hormones. Accretion of fat (and therefore increased leptin) has been shown to promote hypothalamic gonadotropin releasing hormone pulse generator activity [34,35], and, thereby, attenuate gonadal feedback suppression of luteinizing hormone secretion [36] and augment aromatase activity in the ovarian granulosa cells [37]. However, our findings, which were largely independent of BMI at 20 years of age, provide evidence that some independent direct effect, acting through additional biological pathways, may be at play.

Earlier menarche was associated with higher levels of insulin like growth factor (IGF)-I, androstenedione, dehydroepiandrosterone sulfate (DHEAS), leptin, and fasting insulin, and with lower levels of IGF binding protein-I, and sex hormone binding globulin (SHBG) at age 8 years in a study of 329 girls [38]. IGF-I, androstenedione, and DHEAS remained associated with earlier menarche after adjustment for BMI and height, suggesting independent functional roles of these hormones, which have been associated with low birth weight and early catch up weight gain, in regulating puberty timing in girls [38].

Insulin-resistance induced hyperinsulinemia manifesting early in life may be an important pathologic perturbation contributing to the observed association between earlier age at menarche and higher diabetes risk. Randomized controlled trials in low-birth weight, precocious-puberty girls have shown that administration of metformin, an insulin-sensitizing medication commonly used to treat type 2 diabetes, delayed age at menarche and improved post-menarcheal (up to 15 years of age) insulin resistance, inflammation, liver fat content, and other pernicious metabolic parameters [39-42]. Further research from cell, animal, and human experiments is needed to shed etiologic light on the interplay of adiposity, insulin resistance, inflammation, the IGF axis, SHBG, and sex hormones, among other biologic intermediaries in the association of earlier menarche with diabetes.

A novel finding from our study was the association between earlier menarche and elevated alanine aminotransferase, triglycerides, and high sensitivity C-reactive protein. This is the first evidence, to our knowledge, linking menarcheal age with markers of liver dysfunction and inflammation. One hypothesis is that these associations are driven by an underlying association between age at menarche and non-alcoholic steatohepatitis–otherwise known as the hepatic manifestation of the metabolic syndrome [43]. While studies finding that early maturing women had higher risk of the metabolic syndrome and its components provide indirect support for this hypothesis [44-47], observational and experimental research is needed to directly examine the potential link of pubertal timing with liver steatosis and inflammation.

This current study took advantage of a rich database in a unique population to study the role of age at menarche in relation to adult diabetes and other cardiometabolic disease risk factors. The fact the association was little changed after adjustment for participant age at enrollment, and factors related to menarcheal age and type 2 diabetes that may vary across birth cohorts, such as prevalence of paternal diabetes, education, birth weight, and early adulthood BMI, makes it very unlikely that the association between age at menarche and diabetes is due merely to a correlation between secular trends of increasing diabetes prevalence and decreasing age at menarche in Brazil.

An important limitation to consider when interpreting the results of this analysis was the self-recall of menarcheal age many years after the event; thus, misclassification was inevitable. However, adulthood retrospective reports of age at menarche are highly correlated (r = 0.79) with original adolescent reports [28]. One might also consider the cross-sectional nature of the data collection a limitation. Yet, in our cohort, all diabetes diagnoses occurred after menarcheal onset, and were therefore incident in relation to age at menarche. We cannot rule out the potential for residual confounding by factors related to both age at menarche and type 2 diabetes. For example, ELSA-Brasil does not have childhood anthropometry (e.g., BMI) or physiologic measures. Childhood fatness and related hormones may cause early puberty and diabetes. To address this concern, we adjusted for BMI (based on self-reported weight) at age 20 years which, based on evidence [48] that BMI tracks well through life, may proxy pre-pubertal BMI.

Understanding how factors along the life course impact type 2 diabetes and cardiometabolic disease risk, and how such factors can be cost-effectively and sustainably modified, are among the transcendent public health challenges of our time. These challenges are especially critical for population health in low- and middle-income countries, including those in South America, in which changing demographics, in addition to behavioral and environmental risks, are producing rapid increases in diabetes, and where the alternative to risk factor prevention–the widespread use of costly drug- and device-related interventions–is neither practical nor cost-effective. As such, beyond its potential for extending quality years of life, investigation into the primordial prevention of type 2 diabetes and other cardiometabolic diseases is critically important to the future economic viability of health care globally.

## Conclusion

These findings support the hypothesis that earlier age at menarche (in our study, less than 11 years) is associated with higher risk of adult diabetes and cardiometabolic disease risk factors, permitting the possibility that pubertal timing plays an independent role in the developmental origins of adult chronic disease. This research provides a platform for exploring the myriad behavioral and environmental factors that may alter pubertal timing landmarks, such as menarche, and thereby increase cardiometabolic disease risk. Continued research in this primordial-prevention realm may hold promise for curbing the rise in metabolic and cardiovascular diseases in low- and middle-income countries like Brazil.

### Consent

All local ethics committees approved the study, and all participants provided written, informed consent prior to entering the study.

## Abbreviations

BMI: Body mass index; OGTT: Oral glucose tolerance test; A1C: Glycated hemoglobin; ALT: Alanine aminotransferase; SHBG: Sex hormone binding globulin; IGF: Insulin like growth factor; HDL: High density lipoprotein cholesterol; CRP: C reactive protein; DHEAS: Dehydroepiandrosterone sulfate.

## Competing interests

The authors declare they have no competing interests.

## Authors’ contributions

NTM designed the analytic strategy, undertook analyses, interpreted results, and wrote, reviewed and edited the manuscript. BBD, AOO, EMA, SMB, DC and MS contributed to the study design, results interpretation and manuscript revision. All authors read and provided final approval of the manuscript.
